# Protein-Based Nanoparticles as Drug Delivery Systems

**DOI:** 10.3390/pharmaceutics12070604

**Published:** 2020-06-29

**Authors:** Seyoung Hong, Dong Wook Choi, Hong Nam Kim, Chun Gwon Park, Wonhwa Lee, Hee Ho Park

**Affiliations:** 1 Department of Biotechnology and Bioengineering, Kangwon National University, Chuncheon 24341, Korea; syh@kangwon.ac.kr; 2 Department of Cancer Biology, Dana-Farber Cancer Institute, Harvard Medical School, Boston, MA 02215, USA; dongw_choi@dfci.harvard.edu; 3 Center for BioMicrosystems, Brain Science Institute, Korea Institute of Science and Technology (KIST), Seoul 02792, Korea; hongnam.kim@kist.re.kr; 4 Department of Biomedical Engineering, SKKU Institute for Convergence, Sungkyunkwan University (SKKU), Suwon 16419, Korea; 5 Biomedical Institute for Convergence at SKKU (BICS), Sungkyunkwan University, 2066 Seobu-ro, Jangan-gu, Suwon 16419, Korea; 6 Aging Research Center, Korea Research Institute of Bioscience and Biotechnology, Daejeon 34141, Korea

**Keywords:** protein nanoparticle, drug delivery, biocompatible, biodegradable, controlled release

## Abstract

Nanoparticles have been extensively used as carriers for the delivery of chemicals and biomolecular drugs, such as anticancer drugs and therapeutic proteins. Natural biomolecules, such as proteins, are an attractive alternative to synthetic polymers commonly used in nanoparticle formulation because of their safety. In general, protein nanoparticles offer many advantages, such as biocompatibility and biodegradability. Moreover, the preparation of protein nanoparticles and the corresponding encapsulation process involved mild conditions without the use of toxic chemicals or organic solvents. Protein nanoparticles can be generated using proteins, such as fibroins, albumin, gelatin, gliadine, legumin, 30Kc19, lipoprotein, and ferritin proteins, and are prepared through emulsion, electrospray, and desolvation methods. This review introduces the proteins used and methods used in generating protein nanoparticles and compares the corresponding advantages and disadvantages of each.

## 1. Introduction

**Figure 1:**
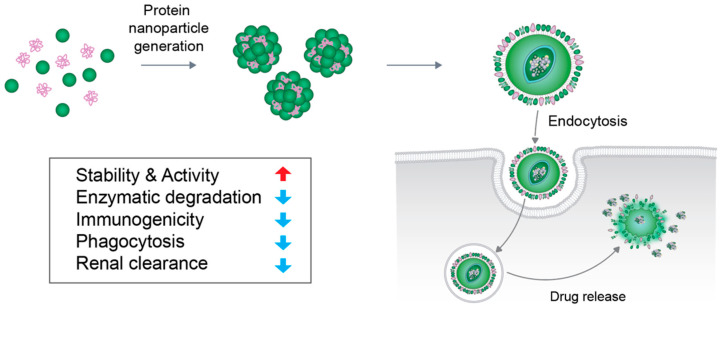
Delivery of protein nanoparticle to the cell. Intracellular delivery of insoluble drugs by protein nanoparticles via the endocytosis process. Protein nanoparticles have several advantages as a drug delivery system, such as increased stability and activity due to increased protection from enzymatic degradation, immunogenicity, phagocytosis, and renal clearance, thereby leading an increase in the half-life of the drug.

**Figure 2:**
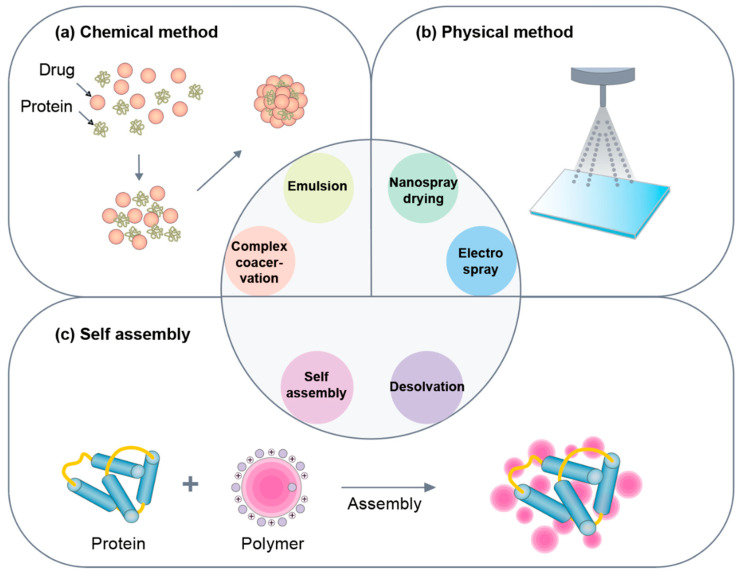
Categorization of methods for preparing protein nanoparticles. (**a**) The chemical method produces protein nanoparticles using a chemical reaction and includes emulsion and complex coacervation. (**b**) The physical method includes physically aggregating after separating proteins into nanosized particles and includes an electrospray technique and a nano spray drying method. (**c**) The self-assembly method is a method of making nanoparticles by agglutinating proteins by themselves and it includes the desolvation method.

The drug delivery system comprises the administration and delivery of pharmaceutical compounds, such as therapeutic drugs, to a specific area in the body with improved efficacy and safety. It involves increased therapeutic effect via increased pharmacokinetics, extension of controlled release, and localized delivery, and release of the drug [1]. Successful treatment can be expected only when the concentration of the drug is maintained at proper levels in the blood and the physiologically active substances are released at a certain rate. Thus, drug delivery systems are becoming increasingly important. Among them, several nanomaterials, such as liposomes [2,3], polymers [4,5], dendrimers [6,7], and magnetic nanoparticles [8,9], are used as carriers for drug delivery. The transport of insoluble drugs via nanoparticles is improving because of their small particle size, which quickly dissolves in the bloodstream and can reach a cell or tissue-specific target. Recently, biopolymer-based nanoparticles, such as protein nanoparticles, have been actively used as pharmaceutical and functional tools owing to their low toxicity and biodegradability, as exhibited throughout the many studies that have been conducted on the subject [10]. Proteins exhibit unique functions and properties in biological materials and manufacturing fields and can be used as base materials for production of nanoparticles [11]. Owing to their small size, protein nanoparticles can be transmitted through the cell via endocytosis [10]. Protein nanoparticles have several advantages as a drug delivery system, such as biodegradability, stability, surface modification of particles, ease of particle size control, and they have less problems associated with toxicity issues, such as immunogenicity [12]. In particular, its stability, activity, and half-life can be improved by protecting the drug from enzymatic degradation and renal clearance. Protein nanoparticles can likewise be used in a variety of targeted therapies, such as lung delivery [13], cancer therapy [14], tumor therapy [15], and vaccines [16], because of its non-antigenic property [17]. Protein nanoparticles can be incorporated into biodegradable polymers in a microsphere structure for controlled and sustained release (Figure 1). As a drug delivery system, the main purpose of designing nanoparticles is to control the particle size, surface area, and surface characteristics, such that nanoparticles carrying the necessary amounts of drugs can exhibit desired pharmacological activity by releasing active agents to achieve part-specific action. Many approaches have been developed for this purpose [18]. This review highlights the various proteins that are used in generating nanoparticles as well as the ways of preparing them (Figure 2). Additionally, this review aims to introduce the advantages and disadvantages of each protein and method.

## 2. Types of Proteins Used to Produce Protein Nanoparticles

### 2.1. Silk Protein Fibroin

Fibroin is the main protein present in silk fibers, comprising 65 to 85% of the total protein thereof [19]. In general, fibroin, which is commonly extracted from silk produced by the *Bombyx mori* silkworm, is extracted by removing the external sericin via a thermochemical degumming process using Na_2_CO_3_. Considering that the separated fibroin is insoluble, a soluble form termed regenerative fibroin is made by using LiBr or CaCl_2_ [20].

Fibroin is a semi-crystalline structure composed of heavy and light chains [21,22,23]. The heavy chain (45% Gly, 30% Ala, and 12% Ser) comprises 12 major hydrophobic domains linked together by 11 hydrophobic hydrophilic sections. Each hydrophobic domain contains a repetitive sequence of Gly-Ala-Gly-Ala-Gly-Ser and several repetitions of Gly-X (X = Ala, Ser, Thr, Tyr or Val), while the hydrophilic portion can be any amino acid sequence. The heavy chain forms stable antiparallel crystalline β-sheets via intermolecular hydrogen bonds (mainly between Gly and Ala) and van der Waals forces. This structure provides silk fibroin with solid mechanical properties and high tensile strength. The light chain is composed of different proportions of amino acids, i.e., 15% Asp, 14% Ala, 11% Gly, 11% Ser, and traces of cysteine [21,22,23]. The light chain is more hydrophilic and less water-resistant, ultimately contributing to fibroin elasticity. Silk fibroin has been reported to have an isoelectric point (IEP) of pH 7 or lower and a molecular weight (MW) of 83 kDa, however, the size may vary depending on the extraction process and treatment period utilized [21,22,23]. Fibroin is frequently employed for nanoparticle generation due to its flexibility, mechanical strength, good stability, low immunogenicity, biodegradability, and biocompatibility, as well as its large amount and low cost [24,25,26]. The zeta potential of fibroin nanoparticles has a negative charge. The surface, coated with a positively charged polymer, such as PEI, chitosan, or EDC, may be used as a crosslinking agent for the purpose of transforming it into a positive charge [24,25,26]. Various factors, including fibroin MW, crystallinity, encapsulated drug properties, and manufacturing conditions, may affect FNP properties, such as average size, size distribution, surface zeta potential, drug encapsulation, release profile, and stability of particle formation. 

The organic solvent also plays an important role in the formation. Polar protic solvents, such as acetone, methanol, and ethanol, can induce spherical fibroin nanoparticles in aqueous fibroin solutions, while acetonitrile does not form fine particles and, instead, forms a fibroin mass without a specific shape [27]. Nanoparticles using fibroins have different particle sizes with a relatively narrow distribution of size (less than 0.5 multivariance index) and produce nanoparticles larger than the actual MW of fibroins with much larger particle sizes. Moreover, higher multivariance indices are produced if the ratio between the initial fibroin concentration and fibroin solution and ethanol is higher [25,28]. In drug encapsulation and release profiles, fibroin crystallinity plays an important role. Fibroin crystallinity is influenced by salt concentration, organic solvent, and temperature. At relatively low salt concentrations, the hydrogen bonding of the crystalline β-sheets becomes loose, thereby resulting in the formation of an irregular structure; however, it can induce fibronectin precipitates as the salt concentration increases. Organic solvents reduce fibroin surface charges through dehydration, thereby increasing crystalline moieties through intramolecular and intermolecular interactions. They alter the non-covalent interactions of secondary structures which increase the crystallinity. High temperature reduces the order of water molecules (i.e., by increasing the water entropy), thereby reducing the solvation of the hydrophobic region which confers higher chances to form more non-covalent bonds. It has also been found that the drug-loading dose depends on the pKa and the solubility of the captured drug. Drug molecules are primarily associated with fibroin through electrostatic interactions, hydrogen bonding, and/or hydrophobic interactions. Furthermore, the storage temperature greatly affects the physicochemical stability of the nanoparticles even in the form of freeze-dried powder. Higher temperature (i.e., 25 °C) causes particle agglomeration, whereas fibroin nanoparticles are stable for more than six months at lower temperatures (i.e., 4 °C) due to less intermolecular and intermolecular interactions. Particle surface properties also affect stability [24,25]. For instance, using a similar desolvation method, particles with a surface charge of less than ± 30 mV tend to aggregate more than particles with a higher charge.

Since fibroin nanoparticles can overcome some disadvantages of low-molecular-weight drugs, many studies have been conducted to deliver and utilize them as a drug delivery system. All fibroin nanoparticles loaded with small molecule drugs display improved drug treatment efficiency, high capture efficiency, controllable sustained release profile, increased drug solubility and stability, drug degradation inhibition, and toxicity reduction. In a recent study, fibroin nanoparticles with an indocyanine green have been developed using supercritical fluid technology [29]. The particles showed high photothermal stability and pH reactions, during which the dye was specifically released from the tumor acidic environment. In addition, these particles were able to destroy tumor cells with hyperthermia caused by light in both in vivo and in vitro scenarios. Natural compounds have been highlighted for their ability to treat various diseases, such as cardiovascular disease [30]. However, due to their low solubility and potential effects on systemic metabolism, they are less therapeutic.

To this end, fibroin nanoparticles have been appreciated for their ability as a drug delivery system that successfully contains numerous natural compounds. Pham et al. produced nanoparticles containing alpha mangosteen, a chemotherapeutic agent that can be extracted from mangosteen pericarp by crosslinking reactions for anticancer purposes [24]. The crosslinking agent, N-ethyl-N-(3-dimethylaminopropyl)-carbodiimide (EDC) or polyethyleneimine (PEI), was used, which led to the production of spherical particles having an average size of approximately 300 nm. The particle surface charge was controlled from −15 to +30 mV by changing the type and amount of the crosslinker. This study confirmed that the surface charge can be controlled through a crosslinking agent in the production of fibroin nanoparticles. The crosslinked nanoparticles showed higher drug entrapment efficiency (70%) and drug loading (7%), as compared with uncrosslinked nanoparticles. In addition, these particles exhibited an ability to increase the solubility of alpha mangosteen, sustain release for more than 72 h, reduce drug hematopoietic toxicity by 90%, and maintain the drug treatment effect. Lozano-Pérez et al. encapsulated and delivered natural antioxidant quercetin with fibroin nanoparticles using a desolvation method [31]. Depending on the ratio of quercetin and fibroin, up to 70% encapsulation efficiency was observed. After controlled release, the activity of quercetin was maintained. A natural anti-inflammatory compound, resveratrol, was delivered using fibroin nanoparticles by having it administered onto a rat colitis model [32]. Fibroin nanoparticles demonstrated non-cytotoxic as well as immunomodulatory properties via the inhibition of the activity of lipopolysaccharide-stimulated RAW 264.7 macrophages (i.e., nitrite production). Studies regarding the rat colitis model showed that resveratrol-loaded fibroin nanoparticles exhibit a better anti-inflammatory effect, as compared with pure resveratrol, thereby suggesting a synergistic effect on the anti-inflammatory action of both fibroin and resveratrol. These studies have demonstrated that fibroin nanoparticles containing natural compounds have a synergistic effect on drug activity. Fibroin nanoparticles can immobilize enzymes through the chemical interactions between fibroin tyrosine amino acid residues and enzyme structures, thus, enzyme stability can be significantly increased while activity is maintained. For example, Kim et al. recently developed a fibroin nanoparticle encapsulated with cationic lipids that is able to bind to the Pin1 isomerase (phosphorine-proline or phosphothreonine-proline motifs of multiple proteins) [33]. Rollyl cis-trans isomerase was delivered directly into the cytoplasm. These fibroin nanoparticle-lipid complexes successfully delivered enzymes with high efficiency and low cytotoxicity, thereby resulting in increased Runx2 and Smad signaling and leading to recovery of the bone formation marker gene expression and deposition of minerals in Pin1-deficient cells. Recently, fibroin nanoparticles have also been highlighted for their versatility, non-toxicity, high transfection efficiency, and DNase resistance properties. Song et al. manufactured a c-myc anti-sense oligo deoxyneucleotide-containing fibroin-PEI NP, with or without the addition of magnetic NP, for targeted delivery to MDA-MB-231 breast cancer cell lines [8]. The particle size and zeta potential were controlled by varying the amount of fibrin. Fibroin-PEI NP was less cytotoxic than PEI NP, thereby enabling the successful transfer of encapsulated genetic material to the MDA-MB-231 cells. Fibroin-PEI combined with magnetic NP using magnetofection showed significantly improved efficiency of DNA delivery within 20 min, as compared with those using non-magnetofection [8]. Shahbazi et al. prepared oligo-chitosan-fibroin nanoparticles for delivery of siRNAs. The particle size was similar to that of polyplex (250 to 450 nm). Furthermore, fibroin concentration-dependent increase in the siRNA loading efficiency was observed [34]. Moreover, increased stability was observed in the presence of fetal bovine serum and heparin, as compared with the fibroin-free polyplex. Fibroin nanoparticles showed decreased cytotoxicity, however, lower siRNA gene silencing efficiency was observed due to the lower loading efficiency.

Fibroin has many advantages as a drug delivery system; however, it still has disadvantages to overcome. Since sericin can cause an immune function, the process of removing sericin from silk fibers must be properly completed [35]. In addition, the slow degradation of the fibroin crystalline antiparallel β-sheet domain can be a disadvantage in terms of some applications that require fast and complete removal of nanoparticle carriers (Table 1). Moreover, fibrin, as a protein, can be subjected to proteinaceous attack by immune systems, such as macrophages and giant cells, and consequently, encapsulation and formation of granuloma inside these cells can lead to drug release outside the target. Finally, since fibroin can be extracted from a variety of sources, which can be similar to other natural products, the nature of each batch could be slightly different due to post-conversion process changes in both species and individuals.

### 2.2. Human Serum Albumin

Human serum albumin (HSA) is a globular protein found mainly in the circulatory system composed of up to approximately 585 amino acids, which is equivalent to a MW of 66 kDa [12]. HSA consists of main units I, II, and III, with each unit containing subunits A and B. Within the HSA, there are two main binding sites known as Sudlow’s sites I and II, located in subunits IIA and IIIA, respectively [36]. It is well-known that HSA acts as a carrier for other substances and enhances the solubility of hydrophobic molecules in the blood. HSA is used to deliver various substances to specific tissues in the body. Furthermore, HSA is very stable against pH (stabilized in the pH range of 4 to 9), temperature (can be heated at 60 °C for up to 10 h) and organic solvents [37,38,39]. It is also of biological origin; thus, it is advantageous in terms of biodegradability, non-toxicity and non-immunogenicity, and high solubility. These advantages make wide use of bovine and HSAs in studies on protein binding and targeted drug delivery applications. Since HSA has a high affinity to various drugs, it is possible to effectively integrate these compounds using a matrix of HSA nanoparticles [40,41] (Table 1). Since functional groups (i.e., amino and carboxy groups) exist on the surface of the nanoparticles, it is possible to co-induce the drug-targeting ligand to the HSA nanoparticles. Moreover, protein nanoparticle preparation, particularly HSA, appears to be a suitable material for drug delivery because it can avoid unwanted interactions with serums that are often encountered after intravenous injection of complexes [38]. HSA nanoparticles have been extensively used in various applications, such as for the treatment of cardiovascular-associated pathologic calcifications in the media of large- and medium-sized arteries. [42].

HSA can bind to a variety of drugs and peptide compounds through non-covalent interactions. Reactive groups, such as amino, thiol, and carboxyl, on the nanoparticle surface, facilitate covalent ligand binding and surface modification. HSA is known to have excellent ligand-binding properties and can be used to load a variety of drugs for delivery via the circulatory system [43,44]. HSA has both high stability and high binding affinity sites for the loading of therapeutic drugs with high concentrations [43,45]. HSA has a binding capacity to seven long chain fatty acids at several binding sites with different affinities [46]. HSA nanoparticles have different characteristics, such as size and polydispersity, depending on production conditions [47]. Jahanban-Esfahlan et al. prepared HSA nanoparticles using the desolvation method and compared the particle size according to various factors, such as the amount of crosslinking agent, the presence of salt, and protein concentration [36]. At a protein concentration of 50 to 60 mg/mL, the amount of crosslinking agent was confirmed to be small (approximately 3 to 5 mg), and when the salt was added to the albumin solution, the formation of solid yellow bulk and the size of the prepared particles were considered to be large. It was confirmed that phosphate buffer and sodium chloride were not suitable materials to produce albumin nanoparticles with a small particle size. It is important to note that the crosslinking agent EDC was used, rather than glutaraldehyde (GA), in this study. In addition, Langer et al. produced HSA nanoparticles using the desolvation method and confirmed the particle size according to the production conditions [12]. It was confirmed that the smaller the ethanol addition rate, the smaller the nanoparticles were formed. The pH of the solvent also affected the particle size and zeta potential; The proper particle size and the zeta potential were achieved at high pH levels. The concentration of the protein was the same as that in the study by Jahanban-Esfahlan et al. described above, the smallest nanoparticles were produced at 50 mg/mL. However, when GA was used as a crosslinking agent, the particle size was not significantly different from that of EDC. Albumin nanoparticles display diverse properties depending on various production conditions. Moreover, HSA nanoparticles are non-toxic, non-immunogenic, and biodegradable, and have several potential binding sites for drugs, making them effective as carriers for anticancer drugs. Saleh et al. prepared HSA nanoparticles containing curcumin using a desolvation method and delivered them to HER-2 positive breast cancer cells in which the size of the nanoparticles was 246.1 ± 15.4 nm and the zeta potential value was −25 ± 2.7 mV [14]. The drug-loading efficiency of nanoparticles was 3.4% and encapsulation efficiency was 71.3%, thereby exhibiting increases in both stability and solubility of curcumin. Furthermore, targeting was possible through surface modification of nanoparticles that were HER2 Apt conjugated to the nanoparticle surface. After the approval of abraxane (albumin-paclitaxel nanoparticle), clinical trials using abraxane have been conducted and these have showed substantial clinical activity among advanced pancreatic cancer patients [48], manageable safety profile with antitumor responses in metastatic triple-negative breast cancer patients [49], and antitumor activity in women with ERBB2/HER2-negative breast cancer [50]. HSA nanoparticles have likewise been studied as carriers for delivering antibodies or genes. Recently, Mesken et al. conducted a study on the delivery of HEK 293T where a plasmid was loaded onto HSA nanoparticles conjugated with cell-penetrating peptide (CPP) [51]. The nanoparticles were prepared using the desolvation method. The particle size was 207.8 ± 21.3 to 222.8 ± 42.4 nm and the zeta potential was −44.7 ± 9.7 mV. Plasmid loading efficiency was found to be 78.3 ± 13.0%, thereby confirming that the surface modification of nanoparticles using CPP did not affect the plasmid loading efficiency. Nanoparticle-mediated transfection displayed little cytotoxicity and no significant difference in efficiency when it was tested at a low plasmid concentration level, while using a high concentration level of the plasmid resulted in increased transfection efficiency of up to 50%. Since HSA nanoparticles are barely cytotoxic, they can also be used as non-DNA carriers. For example, Redín et al. developed HSA nanoparticles loaded with bevacizumab, a chemical drug used for treating cancers and a specific eye disease [52]. The payload of the nanoparticles generated using the desolvation method was improved by increasing antibody to albumin ratio to 0.15. Under these experimental conditions, the resulting bevacizumab nanoparticles exhibited an average size (close to 300 nm) with zeta potential of about −15 mV. The nanoparticles exhibited high stability and exhibited a two-phase release pattern characterized by an initial release of about 400 μg/mL during the first 5 min, followed by a slower and more sustained release rate for more than 24 h. In addition, it was confirmed that albumin nanoparticles did not show toxicity in vivo and showed mucosal adhesion. Albumin nanoparticles have also been proposed as a good carrier for antibody delivery.

### 2.3. Gliadin

Gliadin is one of the main proteins in wheat gluten and is extracted using 70% ethanol. Gliadin consists of single-chain polypeptides linked by intramolecular disulfide bonds, with each polypeptide chain having an average MW of 25 to 100 kDa [53]. Gliadin has low solubility in aqueous solutions, except for extreme pH. This low water solubility is due to the hydrophobic interactions and disulfide bonds that lead to a distinct folded structure of the protein. Because of this, gliadin nanoparticles can be used as a controlled-release system suitable for hydrophobic and amphiphilic drugs. The advantage of using water-insoluble proteins is that no additional curing step is required to maintain the integrity in water-based products. Hence, gliadin has excellent biocompatibility, biodegradability, non-toxicity, and stability, and is suitable for use as a drug delivery system. In addition, because it attaches to the mucosa, it can be a polymer suitable for oral and local drug delivery systems that can be affixed to the mucous membrane [53] (Table 1). Gliadin nanoparticles are polymers suitable for targeted drug delivery because they show affinity for the upper gastrointestinal tract, but are not well anchored to other gastrointestinal tracts [54]. For example, Umamaheshwari et al. developed mucoadhesive gliadin nanoparticles containing amoxicillin to eradicate *Helicobacter pylori* [55]. The gliadin nanoparticles were prepared using the desolvation method, and the size ranging from 392 ± 20 nm to 285 ± 44 nm showed a zeta potential of 26.6 ± 0.8 mV. The payload of gliadin nanoparticles was about 60%. The size of the nanoparticles was proportional to the concentration of gliadin. When nanoparticles were delivered in vivo, *Helicobacter pylori* was more effectively eradicated, compared to free amoxicillin in the gastrointestinal tract, due to prolonged residence time as a result of mucosal adhesion. The hydrophobicity and low solubility of gliadin can produce nanoparticles that protect the loaded drug and control its release. Gulfam et al. used gliadin nanoparticles to induce the death of breast cancer cells [56]. They used an electrospraying system to generate gliadin and gliadin-gelatin complex nanoparticles to control the release of the anticancer drug cyclophosphamide (CP). Gliadin nanoparticles containing CP were released within 48 h, while nanoparticles composed of gliadin-gelatin showed rapid emissions. The size of the nanoparticles was 218.66 ± 5.1 for the gliadin nanoparticles and 398.56 ± 4.2 for the gliadin complex nanoparticles, while the drug loading efficiency was higher at 72%. Gliadin is rich in neutral and lipophilic amino acid residues. Furthermore, it was shown that the neutral amino acids promote hydrogen bonding with the mucosa while the lipophilic amino acids interact with biological tissues through hydrophobic interactions. Ezpeleta et al. prepared a gliadin-based nanocarrier system for the administration of trans-retinoic acid (RA) [54]. Gliadin nanoparticles containing RA were prepared using the desolvation method and showed a size of 500 nm. This method can produce gliadin nanoparticles with a yield of 90% of the initial protein. These nanoparticles were stable in phosphate buffered saline (PBS) at pH 7.4 for 4 days. However, gliadin nanoparticles are susceptible to pH, heating, and salt, thereby resulting in aggregation and instability [57]. Recently, several studies have shown that protein-polysaccharide interactions can be used to improve the stability of protein nanoparticles against environmental stress [58,59]. Gum arabic (GA) stabilized multiple proteins due to higher receptivity and lower viscosity, as compared with other polysaccharides [60,61]. Wu et al. added GA to increase the stability of the gliadin nanoparticles. The GA solution had a negative zeta potential and the absolute value increased as the pH increased [53]. As the ratio of gliadin and GA (4:1 to 1:3) decreased, gliadin/GA exhibited a negative zeta potential and the absolute value increased with increasing pH. This showed that the addition of GA can improve the stability of gliadin/GA particles at pH, and that the higher the ratio of GA, the greater the stability improvement was observed. Gliadin nanoparticles increased in size as the salt concentration increased and the pH decreased. Moreover, the gliadin/GA nanoparticles also increased in size, but showed smaller sizes than gliadin nanoparticles. This confirmed that gliadin/GA nanoparticles were also affected by salt concentration and pH. In addition, the gliadin/GA nanoparticles were hardly deformed after heating at 80 °C for 1.5 h, thereby indicating that it is caused by heat-induced aggregation.

### 2.4. Gelatin

Denatured protein gelatin is a natural water-soluble polymer that can be obtained by hydrolyzing collagen in alkaline or acidic mediators or by thermal or enzymatic degradation of collagen. Furthermore, it is the earliest proteinaceous material used in the formulation of nanoparticles [62]. The average size of gelatin is 20 to 220 kDa, and it is a soluble protein in water at above 35 to 40 °C [63]. Commercially, two different types of gelatin (type A & type B) are available depending on the method of collagen hydrolysis [64,65,66]. Cationic gelatin (type A with an IEP of 7 to 9) is derived from partial acid hydrolysis of pig skin type 1 collagen, while anionic gelatin (type B, IEP of 4.8 to 5) is extracted from alkaline bovine collagen. Gelatin contains hydrophobic groups, both cationic and anionic groups, and a three-helix structure with repeating sequences of glycine, proline, and alanine [16,67]. The three polypeptide α-chains in the three-helix structure enable high stability of gelatin. Commercially available gelatin is a heterogeneous mixture consisting of 18 specific amino acid polypeptide chains. The primary structure of gelatin enables various chemical modifications for instance drug attachment via its covalent bond. This can be achieved via the matrix or the surface of gelatin particles [68]. In the case of the matrix, chemical modification is required on the gelatin macromolecule before the nanoparticles are formed. These properties, combined with the high potential of nanosized delivery systems, enable gelatin-based nanoparticles to be a promising carrier system for drug delivery [69]. As gelatin is commercially available, Food and Drug Administration (FDA)-approved, and a generally-recognized-as-safe protein, it is used, through intravenous infusion, as a food supplement and plasma expander. Gelatin nanoparticles are widely used to encapsulate DNA and RNA, as well as several biologically active molecules, such as bovine serum albumin (BSA), bone morphogenetic protein 2 (BMP-2), and basic fibroblast growth factor (bFGF). However, gelatin has low mechanical strength and rapid decomposition rate in terms of nanoparticle generation (Table 1). Therefore, when nanoparticles are manufactured using gelatin, it must be physically, biologically or chemically crosslinked by various crosslinking agents, such as GA to increase mechanical strength and lower the decomposition rate and solubility in aqueous solutions. It is also necessary that it is crosslinked physically, biologically or chemically by various crosslinking agents [1]. Wang et al. conducted a study in which gelatin nanoparticles loaded with BMP-2 and gelatin nanoparticles loaded with bFGF were prepared, mixed, and delivered to bone in vivo. Nanoparticles were prepared using the desolvation method. GA was used as the crosslinking agent, and various crosslinking densities were used [70]. The rate of drug release was different depending on the crosslink density. The higher the crosslinking density, the slower the decomposition rate. Furthermore, it was loaded into nanoparticles that rapidly decomposed at a low crosslinking density that showed lower resolution at a high crosslinking density. The mixture of nanoparticles showed higher efficiency in terms of bone regeneration. This indicates that the drug release from gelatin nanoparticles can be controlled by crosslinking density and can be used for drug delivery. It has been confirmed that gelatin nanoparticles can be surface-modified to transport DNA and proteins through ionic interactions. Cationized gelatin is mainly prepared by introducing amine residues into the carboxyl groups of gelatin using polyethyleneimine [71], cholamine [72], ethylenediamine [73], spermidine [74], and spermine [75]. The main characteristic of gelatin nanoparticles is the ease of accumulating in macrophages and macrophage-rich organs and crossing the blood-brain barrier. It is widely used as a vector for the delivery of various anticancer drugs, herbal extracts, and therapeutic biopolymers. Chou et al used PEI to cationize the surface of gelatin nanoparticles and deliver proteins into cells [76]. The nanoparticles were prepared using desolvation method, and the zeta potential of the nanoparticles was approximately +60 mV and the particle size was about 135 nm. The particles were stable at different biological pH values and temperatures, and high protein loading efficiency was observed. In addition, it was confirmed that the protein was accumulated through cellular uptake and that it showed no cytotoxicity. Therefore, PEI-modified gelatin particles can be used as a biodegradable and highly efficient protein delivery system.

### 2.5. Legumin

Legumin is one of the major storage proteins of soybean seeds (*Pisum sativum* L.) and it belongs to the 11S globulin protein family. Legumin has a molecular mass of 300 to 400 kDa, is rich in sulfur-containing amino acids, and consists of six subunits [77]. The nanoparticles derived from legumin are bioadhesive and have a large surface area, thereby indicating high interaction potential with biological surfaces [78]. The coacervation method has been the most frequently applied method in terms of the synthesis of legumin nanoparticles. The solubility of legumin decreases during the coacervation process and induces phase separation to form nanoparticles. Mirshahi et al. attempted to manufacture micro- and nanoparticle-formed legumin colloidal delivery systems to achieve sustained release and targeted delivery of the drug [79]. After aggregation, nanoparticles were formed through chemical crosslinking using GA. Chemical crosslinking of the pH-coacervation method and GA has been attempted to obtain good yield, size, and surface charge while avoiding the use of organic solvents. Nanoparticles were very stable in PBS (pH 7.4), however, this method yielded nanoparticles as low as 27% of the original materials (Table 1). Crosslinking with GA decreased the antigenic determinants of legumin, leading to the reduction in the immunogenicity of the protein [80]. The optimum pH for obtaining submicron size coacervates is near neutral. The size of the particles ranges from 250 to 300 nm at pH 4.5 to 7. The particles showed good stability when stored under neutral pH conditions. Legumin-based nanoparticles display small size, excellent stability, and low antigenicity; however, more research on optimization is necessary to improve the low yield associated therewith and to establish their usefulness in biomedical applications of legumin nanoparticles.

### 2.6. 30Kc19 Protein Derived from Silkworm Hemolymph

The 30K protein family (30Kc6, 30Kc12, 30Kc19, 30Kc21, 30Kc23) is a group of proteins derived from the hemolymph of silkworms (*Bombyx mori*) that have similar structures [81]. The MW of the 30K proteins is about 30 kDa, it plays roles in cell growth and viability in various cells, and has an enzyme-stabilizing effect [82,83]. It has been shown that 30Kc19, the most abundant protein in the 30K protein family, has a cell-penetrating effect [84]. The 30Kc19 protein consists of six alpha-helixes of the N-terminal domain and 12 beta-strands of the C-terminal domain [85]. The CPP Pep-c19 is located in the α-helix domain [84,86]. Lee et al. conducted a study to deliver β-galactosidase (β-gal) into cells using 30Kc19 protein nanoparticles [84]. Nanoparticles were prepared using the desolvation method and then crosslinked using GA. When the nanoparticles were manufactured using only 30Kc19, the nanoparticles were not well-formed and the size was too large. However, when 30Kc19-HSA nanoparticles were prepared with 50 wt% of 30Kc19 and HSA, the particle size was small and the drug activity was high. pH was reciprocally proportional to the concentration of 30Kc19 and the particle size. The loading capacity showed a high yield of 80 to 90% of the initial protein. Using the 30Kc19 protein nanoparticles, 30 to 50% of β-gal was released within 24 h and continuous release up to 60% was observed. They also generated nanoparticles containing α-galactosidase (α-gal) using 30Kc19 and HSA, and successfully delivered them to cells [87]. As the wt% of 30Kc19 protein increased from 0 to 70%, the size of nanoparticles increased from 230 nm to 310 nm. α-gal was loaded with a high yield of 80 to 95% in the 30Kc19 nanoparticles. The specific activity of the α-gal in the nanoparticles was higher than 30 to 50 wt% of 30Kc19 to 20 wt%, thereby demonstrating the enzyme-stabilizing effect of 30Kc19. The morphology of all 30Kc19 nanoparticles was spherical. 30Kc19-HSA nanoparticles showed higher cellular uptake in human fibroblasts, as compared with the HSA nanoparticles.

The α-helix domain of the 30Kc19 protein (30Kc19α domain) is associated with the enzyme-stabilizing effect of the 30Kc193 protein on the cargo protein, and, in particular, the cell-penetrating ability of the 30Kc19α subunit was higher than that of the entire 30Kc19 protein [85]. In addition, the efficiency of intracellular cargo protein delivery of 30Kc19α was almost similar to that of the Pep-c19 CPP. The α-helix domain (30Kc19α) has been shown to exist in a soluble form, while the β-sheet domain (30Kc19β) appeared in an insoluble form. Recently, Park et al. developed 30Kc19α nanoparticles allowing for the successful delivery of β-gal into cells without the requirement of HSA during nanoparticle generation [85]. Protein nanoparticles were made via the desolvation method. Smaller nanoparticle size was achieved at high pH and low concentration of 30Kc19α. Loading capacity was 60 to 65%, which is less than that of the 30Kc19-HSA nanoparticles. β-gal released more than 30% within 10 h, while a sustained release was observed up to 60%.

It has been challenging to produce small-sized nanoparticles using only the 30Kc19 protein. However, 30Kc19-HSA nanoparticles, including 50% of 30Kc19, showed higher activity of the loaded protein and intracellular delivery efficiency, as compared to HSA nanoparticles. These findings suggest that HSA mixed with 30Kc19 is more suitable as a nanoparticle material for drug delivery than conventional HSA. In addition, it was confirmed that 30Kc19α, a 30Kc19 α-helix domain, can be used to make nanoparticles even when used singly, and that the efficiency of intracellular cargo protein delivery was similar to that of Pep-c19 cell-penetrating peptides, thereby suggesting that it can be used as a material for protein nanoparticles.

### 2.7. Lipoprotein

Lipoproteins are natural nanoparticles that transport fats within the body [88]. Many attributes render lipoproteins attractive and versatile delivery vehicles. Various types of lipoprotein nanoparticles exist, all of which have a similar structure with a core composed of triglycerides and cholesterol esters that are covered with a layer of phospholipids with embedded amphipathic apolipoproteins [89]. Lipoproteins can be categorized into several classes according to size and density, ranging from high density lipoprotein (HDL; 7 to 13 nm), low density lipoprotein (LDL; 22 to 27 nm), intermediate density lipoprotein (IDL; 27 to 30 nm), very low density lipoprotein (VLDL; 35 to 80 nm), and Chylomicrons (80 to 1200 nm) [90]. These lipoproteins are characterized by their size, density, lipid composition, major apolipoproteins, and function. In general, lipoprotein nanoparticles are separated from plasma using a density-based ultracentrifugation method [91].

Natural nanoparticles, such as lipoprotein nanoparticles are attractive alternatives to synthetic nanocarriers in terms of the delivery of drugs, due to their biocompatible, non-immunogenic, biodegradable, and naturally targeting properties. Lipoproteins exhibit relatively long circulation half-lives, as compared with non-lipoprotein nanoparticles [92], and the circulating half-life is in the order of 48 to 72 h [93]. Lipoprotein nanoparticles can also be loaded with therapeutic materials, such as drugs [94] and nucleic acids [95], while ligands can be attached onto the surface for targeting purposes [96]. Lipoprotein nanoparticles have been used for the treatment of Alzheimer’s disease [97].

Lipoproteins are known to play important roles in cardiovascular diseases. The plasma levels of LDL-cholesterol have shown a correlation with the risk of coronary artery-related diseases [98]. Reduction in plasma levels of LDL-cholesterol has been shown to lower the risk of coronary artery-related diseases using the statin drug [99]. In addition, the anti-CSK9 (proprotein convertase subtilisin/kexin type 9) antibody against Phave has some promising results in terms of the reduction of LDL levels [100]. LDL is an attractive drug delivery system for certain types of cancer cells because some cancer cells overexpress the LDL receptor and take up LDL at a rate of up to fifty times higher than that of the normal tissue [101]. In the case of HDL-cholesterol levels, they have shown an inverse correlation with the risk of cardiovascular-related diseases [102]. SR-BI, a high affinity HDL receptor is expressed in the liver, adrenals, and in macrophages [103]. Moreover, it has been regarded as a mechanism for HDL-based drug delivery systems to target cancer [104]. Several HDL-based nanoparticles have undergone clinical evaluation. In particular, the expression level of apoA-I Milano (a mutant version of apoA-I) correlated with a decrease in the risk of cardiovascular disease [105]. Weekly administration of apoA-I Milano/phospholipids for 5 weeks significantly decreased the coronary atherosclerosis [106]. 

### 2.8. Ferritin

Ferritin, a protein with iron storage capability, which was first discovered in 1937 by Laufberger, can be found in microorganisms, plants, and animals [107]. It is a hollow globular protein of MW of 474 kDa, comprised of 24 subunits, in which a 6-nm inorganic core of hydrated iron oxide ferrihydrite is surrounded by a spherical polypeptide shell (Apoferritin). There are two classes of ferritin (H and L) in mammalian cells, which function complementarily in relation to one another in the iron absorption process [108]. Twenty-four copies of the same ferritin subunits are self-assembled forming ferritin nanoparticles [109]. The H subunit contains a dinuclear ferroxidase site located within the four-helix, which catalyzes the oxidation of iron by O_2_. The L subunit does not have this dinuclear ferroxidase site, however, it contains extra glutamate residues on the inner surface of the protein shell, which promote mineralization and the turnover of iron (III) at the H subunit ferroxidase site. Iron enters the ferritin nanoparticles via eight hydrophilic pathways across the protein shell [110]. Ferritin nanoparticles have external and internal interfaces, and this unique structure enables targeting and drug loading. The outer surface of ferritin nanoparticles can be chemically modified and a functional motif can be added, while the inner part can be filled with various high affinity small molecules and metals [111,112]. A remarkable property of ferritin is that it is thermostable and can withstand high temperatures up to 75 °C for 10 min. In addition, it is also stable in the presence of denaturants. Moreover, restoration is possible with ferritin nanoparticles when returning to pH 7.5 from protein structure disassembling condition at pH 2.5. 

Liang et al delivered doxorubicin (Dox) using H-ferritin–nanocaged nanoparticles developed a natural H-ferritin (HFn) nanocarrier that specifically delivered a high concentration of the therapeutic drug doxorubicin (Dox) to tumor cells and significantly inhibited tumor growth with a single-dose treatment [113]. It has been reported that HFn nanocages can bind specifically to tumor cells that overexpress transferrin receptor 1 (TfR1) [114]. Dox-loaded HFn (HFn-Dox) specifically bound and subsequently internalized into tumor cells via interaction with overexpressed TfR1 and released Dox in the lysosomes. HFn-Dox exhibited more than ten times higher intratumoral drug concentration than that of the Dox-free group and significantly inhibited tumor growth. Compared with the clinically approved liposomal Dox (Doxil), HFn-Dox exhibited longer median survival times and lower toxicity when administered at the same dose in all tumor models that were studied.

## 3. Methods for Producing Protein Nanoparticles

There are three methods for preparing protein nanoparticles: 1. chemical method, 2. physical method, and 3. self-assembly (Figure 2). As chemical methods, emulsion and complex coacervation methods are frequently used. The physical methods include electrospray technique and a nano spray drying method. The self-assembly method includes the desolvation method. Each method has its own set of advantages and disadvantages (Table 2).

### 3.1. Chemical Method

#### 3.1.1. Emulsion/Solvent Extraction

**Figure 3:**
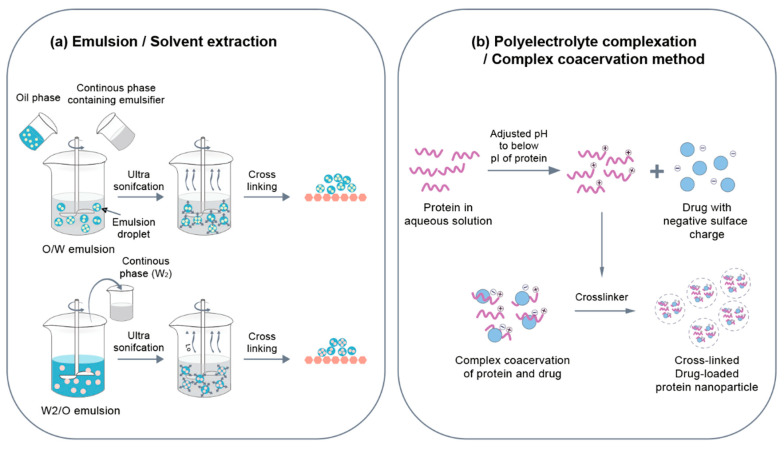
(**a**) The emulsion process is a method of forming nanoparticles by removing solvent/non-solvent after an emulsion system is formed by dispersing via mechanical agitation or ultrasonic waves. Moreover, a double emulsion method was also used. (**b**) The complex coacervation method adjusts the pH to producing the protein cationic or anionic and then interacts with other polymers to produce nanoparticles.

Emulsion/solvent extraction methods are commonly applied to polymer nanoparticles but it has also been also used to produce protein nanoparticles. Emulsion is a mixture of two or more immiscible liquids wherein one or more of the liquids are dispersed into another liquid [115,116]. In its simplest form, a polymer solution (in organic solvent [O]) or protein solution (in aqueous buffer [W]) is dispersed using mechanical agitation or sonication to form an emulsion system (O/W or W/O), followed by the solvent/non-solvent being removed to form nanoparticles (Figure 3a). The use of certain organic solvents, such as ethyl-acetate and chloroform, as well as surfactants, such as poly (vinyl alcohol) and polysorbate-80, is undesirable because it can alter the biological activity of protein therapeutics, thereby possibly resulting in unwanted reactions [117,118]. Recently, a double emulsion method was used to provide high encapsulation efficiency of the payload [119]. In the W/O/W double emulsion method, the primary W/O emulsion is dispersed in a second water phase using a surfactant for stabilizing the emulsion. Thereafter, an emulsion system is formed, and organic solvent O is removed to preserve the nanoparticles in an aqueous buffer (Figure 3a). Synthesis of double emulsions of nanoparticles is a fast and cost-effective method. Emulsification is usually thermodynamically unstable and particle bonding occurs to minimize the free energy of the system [120]. Therefore, a surfactant and a stabilizer are needed to stabilize the emulsion particles. Surfactants are likely to affect drug-matrix interactions and the rate of drug release in a physiological environment. For protein nanoparticles, protein concentration and relative volume ratio of water and oil phases are important parameters. For instance, Mishra et al. prepared bovine serum albumin (BSA) nanoparticles in a W/O fashion [121]. Nanoparticles were prepared in a size range from 100 to 800 nm according to the BSA concentration and the relative W:O volume ratio. Small-sized nanoparticles were realized by increasing concentrations of BSA and the volume of the water phase. The protein nanoparticles formed by this method can be chemically stabilized by adding a crosslinking agent or thermally stabilized and then purified by adding a W/O emulsion to a preheated oil at temperature of 100 °C or higher. Crisante et al. produced BSA nanoparticles using an emulsion method to deliver antibiotics [122]. This method uses dropping of the aqueous phase into an organic phase containing GLA to avoid polymer crosslinking prior to emulsion formation. The loading efficiency of the drug was about 20% and it showed 70% drug release within 48 h. Yang et al. prepared 10-hydroxycamptothecin (HCPT)-loaded BSA nanoparticles using emulsion method. In order to stabilize the nanoparticles, a heat stabilization technique was used rather than a crosslinking agent [115]. Small particle size was achieved by decreasing the concentration of the BSA and the volume of the water phase and increasing the heat stabilization temperature. The drug-loading efficiency of BSA nanoparticles was 57.5%. The drug release was 25% within 48 h, however, 90% of drug release was observed within 20 h when trypsin was treated to simulate in vivo conditions.

#### 3.1.2. Polyelectrolyte Complexation/Complex Coacervation Method

Since proteins are amphoteric with multiple charged functional groups, they can be made cationic or anionic by adjusting various factors such as the pH of the protein. The charged protein can interact electrostatically with other polymeric electrolytes. The pH-dependent electrostatic interaction between proteins and other polymers can be used to design stable biocompatible nanoparticles and coacervates to facilitate the controlled transmission of bioactive treatments and DNA [123]. A cationic protein polymer is commonly used to form a complex with anion oxygen atoms in a phospholipid backbone of oligonucleotides to reduce DNA/RNA therapeutic molecules shaped like long strings to nanoparticles [124] (Figure 3b). For example, Truong-Le et al. captured DNA in gelatin nanoparticles using salt-induced complex coacervations [123]. Gelatin was positively charged at pH 5 and formed a complex coacervate with DNA. The size of the particles ranged from 200 to 700 nm and the loading efficiency was 25 to 30%. During the coacervation process, DNA was physically captured within the protein matrix. Along with electrostatic interactions, hydrophobic interactions or hydrogen bonds were observed to have been capable of improving protein-polymer synthesis. It is suggested that the balance between protein-polymer interaction and polymer-polymer interaction determines the amount of proteins and polymers in the complex. Rhaese et al. generated HSA-PEI-DNA nanoparticles via the complex coacervations process [125]. The desalination was achieved by mixing the HSA solution of pH 4 with PEI and adding a sodium sulfate solution containing DNA. EDC was used as the chemical bridging agent to stabilize nanoparticles to obtain nanoparticles in a size range from 300 to 700 nm. Ren et al. confirmed the effects of ultrasound frequency on the properties of zein-chitosan complex [126]. Resveratrol was successfully encapsulated using zein-chitosan complex coagulation. The effects of ultrasonic waves were confirmed in the manufacturing of nanoparticles wherein 28/40 kHz dual-frequency ultrasound had the highest encapsulation efficiency (65.2%) followed by high encapsulation efficiency (51.1%) at 20/28/40 kHz. Dual-frequency ultrasonic treatment significantly reduced the size of the zein-chitosan complex coacervation particle and reduced their distribution. It also increased thermal stability of nanoparticles without affecting protein structures. Altogether, the size and loading efficiency of nanoparticles can be adjusted through ultrasonic processing when complex coacervates are produced.

### 3.2. Physical Method

#### 3.2.1. Nano Spray Drying

**Figure 4:**
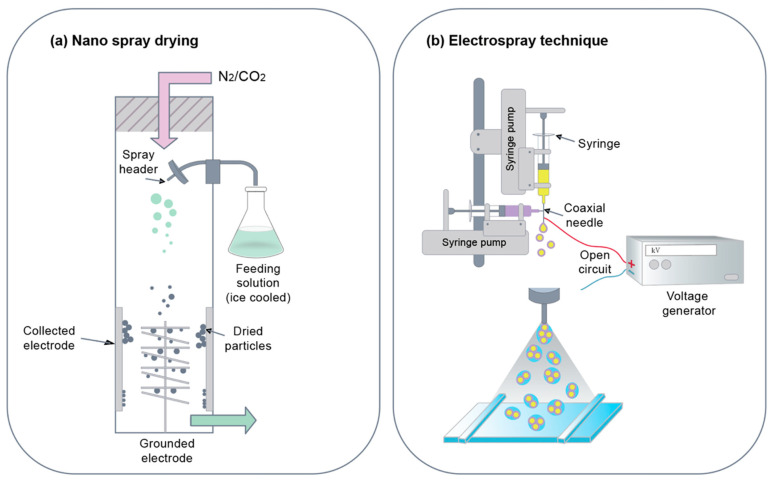
(**a**) Nano spray drying is the process of releasing and drying a protein liquid jet stream with a nozzle using heated nitrogen and carbon dioxide gas to produce nanoparticles. (**b**) The electrospray technique generates nanoparticles by ejecting a liquid jet stream through a nozzle that forms an aerosolized droplet by applying a high voltage to a protein solution supplied through a nebulizer.

Nano spray drying is a technique used in processing nanoparticles in liquid samples. Liquid samples are sprayed into chambers where heated nitrogen and carbon dioxide gas flow in the direction of spray from the nozzle [127]. An electrode at the bottom of the chamber is used to collect nanoparticles. The sprayed droplets are charged electrostatically because of these electrodes, as they move toward the bottom of the chamber. This is a step-by-step process as well as a fast and cost-effective way of producing small-scale protein particles. One application of spray drying is drug delivery systems as hydrophilic drugs can be encapsulated in these spray-dried nanoparticles (Figure 4b). The nanoparticles enable the use of the technique on heat-sensitive specimens as solvent evaporation helps maintain the temperature of nanoparticle droplets [128]. This method of nanoparticles synthesis is beneficial because the size of the particles can be controlled by changing parameters, such as the size of the nozzle and the rate at which the particles are sprayed. In the case of protein nanoparticles, surfactant additions are often required to stabilize polymer particles. Lee et al. produced BSA nanoparticles using Nano Spray Dryer B-90, where Tween 80 was used as a surfactant [129]. The addition of the surfactant changed the shape of the particles to spherical, thereby stabilizing the nanoparticles. Consistency in morphology was achieved at a high concentration of the BSA. The size of the particles was mainly determined by the size of the spray mesh and the BSA concentration.

#### 3.2.2. Electrospray Technique

Electrospraying is a method of liquid atomization. It has been used to manipulate materials into the submicron scale. This method requires the application of high voltage to the protein solution to be able to spray the liquid jet stream through the nozzle for the formation of the aerosolized droplet [56,130]. Aerosolized droplets contain collected colloidal-sized protein nanoparticles (Figure 4a). Using this method, drugs and nucleic acids can easily be integrated into nanoparticles with high efficacy. Solid particles can be produced via solvent evaporation [131]. The needle gauge diameter, applied voltage, flow rate, and operating distance vary depending on the type of drug delivery system. The principle of electrospraying is to apply high voltage to polymer solutions to ensure that polymers come out of syringes in the form of nanoparticles [132,133]. Indeed, electrospraying is a technology similar to electrical radiation used in the production of nanostructures. The advantages of electrospraying include cost effectiveness, reproducibility, and high encapsulation efficiency. Another advantage is the ease for the synthesis and stable protein or carbohydrate polymer nanoparticles without a decrease in encapsulation efficiency and issues related with biocompatibility. The new version of the conventional electrospraying is the coaxial electrospraying method, which uses a coaxial spray head to simultaneously guide both solutions to the electric field. Yang et al. generated gliadin nanoparticles containing meletin using the electrospraying method. The particle size was 570 ± 80 nm, while the drug release efficiency was 28.8% within 1 h of the treatment and 93.7% was released within 16 h [134].

### 3.3. Self-Assembly

#### 3.3.1. Self-Assembly

**Figure 5:**
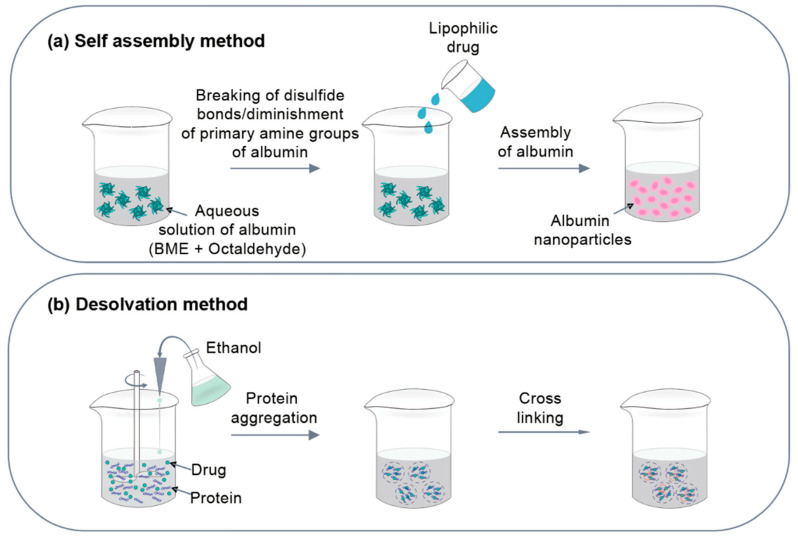
(**a**) In the self-assembly method, individual protein chains are dissolved in an aqueous solution and a CMT exceeding the CMC to spontaneously generate protein micelles during the formation of nanosized aggregates. (**b**) In the desolvation method, nanoparticles are prepared through a simple process of adding a desolvating agent to a protein solution containing drugs.

Protein micelle can be spontaneously produced when individual protein chains are dissolved in a solution exceeding the critical micelle concentration (CMC) and at the critical solution temperature (CMT) to form nanosized aggregates [135] (Figure 5a). By forming a bridge between the chains, micelles can be stabilized via the solidification process. Albumin, a hydrophilic protein, can be given an amphiphilic property through the hydrophobic modification process. Hydrophobically modified proteins can be self-assembled into micelle nanoparticles upon its addition to aqueous solutions. Moreover, hydrophobic cores can serve as a conduit for active molecules. Gong et al. synthesized protein micelle nanoparticles based on the specific reaction between the primary amino group of albumin and octaldehyde and they were able to form a core-shell nanomicelle [136]. It was dissolved in water, integrated, and filtered through a 0.45 μm pore-sized fine porous membrane. The paclitaxel was successfully loaded into the protein micelle through the dialysis method with about 33.1% high drug loading and 90.5% high encapsulation efficiency. Sabra et al. manufactured a zein-lactoferrin (zein-Lf) micelle that highly encapsulated hydrophic drugs, such as rapamycin (RAP) and wogin (WOG), through the hydrophobic coupling reaction [15]. Hydrophilic Lf was included to expand the spectrum of tumor targets. GA was used as a crosslinking agent. Zein-Lf micelle displayed a slow spread of the RAP from the zein core with relatively fast WOG release. This sequential release may lead to efficiency in pump infusion by WOG, thereby sensing tumor cells to RA action. Zein-Lf micelle also showed improved stability and excellent hemocompatibility. Hence, the combined nano-delivery system maximized the synthetic cytotoxicity of RAP and WOG in MCF-7 breast cancer cells.

#### 3.3.2. Desolvation

Desolvation is the most commonly used method in terms of the production of protein-based nanoparticles [68,137]. The desolvation method allows for the synthesis of nanoparticles through a simple process of adding desolvating agents, such as ethanol and acetone, to protein solutions containing drugs. Desolvating agents change the protein structure and reduce the solubility of the protein, thereby leading to the formation of precipitation in the form of protein nanoparticles (Figure 5b). The formation of particles during which the particle size increases up to a certain level and is achieved by a gradual increase in the number of the same size of particles [137]. Once nanoparticles are formed, they are bridged by bridging agents, such as GA. Protein nanoparticles manufactured by the desolvation method can adjust particle size according to conditions [138]. Protein concentration, desolating agent additive speed, pH, and temperature are the main factors affecting particle size. In particular, high pH and low protein concentration can produce smaller nanoparticles. The desolvation method is not directly involved in the synthesis of nanoparticles, but it is a self-assembly method because the protein is concentrated by reducing the solubility of the protein using the desolvating agent. The method is widely used in the generation of nanoparticles using albumin. Langer et al. employed a desoluble agent for HSA receptors at pH levels of 7 to 9 and acetone to generate particles, followed by the stabilization of the particles using GA as a bridging agent [12]. In other studies, parameters, such as the speed of ethanol addition, pH of protein solution, HSA concentration, and refining conditions, were optimized to manufacture HSA nanoparticles with sizes ranging 100 to 300 nm using the desolvation method. Particle size was found to be dependent on the protein concentration, volume, pH, and temperature of the desolvating agents for the volume of protein solution. Lee et al. encapsulated and delivered α-galactosidase (α-gal) into HSA and recombinant 30Kc19 protein nanoparticles [87]. 30Kc19 is a silkworm protein with cell-penetrating and enzyme-stabilizing effects. The nanoparticles were stabilized by the bridging with GA. The nanoparticle diameter was within the range of 230 to 310 nm, depending on the wt% of 30Kc19-HSA, while the loading efficiency was within the range of 80 to 95%.

## 4. Characteristic of Protein Nanoparticles

### 4.1. Particle Size and Polydispersity

Particle size and size distribution are the most important characteristics of nanoparticle systems [139]. Numerous studies have shown that nanoparticles have many advantages over microparticles as drug delivery systems. In general, nanoparticles are applicable to a wide range of biological targets because they display significantly higher intracellular absorption with their smaller sizes, as compared to microparticles, and they are relatively mobile [140]. For example, a body distribution study showed that nanoparticles larger than 230 nm accumulate in the spleen due to capillary size. Other in vitro studies suggested that the particle size of nanoparticles also affects cell absorption. It was also found that nanoparticles coated with Tween 80 were able to pass through the blood-brain barrier, thereby allowing nanoparticles to pass through the blood-brain barrier after a hard joint was opened by the hyper osmotic molecule, suggesting its therapeutic potential in treatment of neurological disorders, such as brain tumors [141]. In some cell lines, submicron nanoparticles can be absorbed efficiently, however, larger-sized fine particles cannot [142,143]. It has been reported that drug emissions are affected by particle size. Considering that smaller particles have a larger surface area, the release of most drugs that are on the particle surface or close to the particle surface is rapid. On the other hand, larger particles can encapsulate more drugs and spread them more slowly. In addition, smaller particles have a greater risk of agglomeration during the storage and transport of nanoparticle dispersion. Formulating nanoparticles of the smallest possible size, but with maximum stability, is always a challenge. Polymer decomposition may likewise be affected by particle size [144]. For example, the rate of poly(lactic-co-glycolic acid) polymer decomposition was found to increase as the particle size increased in vitro. Currently, the fastest and most common method for measuring particle size is measurement using Photon Correlation Spectroscopy (PCS) or Dynamic Light Scanning (DLS) [140]. PCS is the preferred method for industrial submicron mouth analysis. Samples analyzed by PCS devices consist of particles that are well-dispersed in liquid media. Under these conditions, particles continue to have a constant random motion called the Brownian motion and the PCS laser passes through it to measure the speed of this motion. The PCS measures the average particle size and PI of samples. The exact measurement values of the particle size are not more than 0.7 (70%) [140]. DLS theory is a well-established technique for measuring particle size ranging from nanometer to micrometer. This concept takes advantage of the idea that small particles in suspension move in random patterns. Larger particles move slower than smaller particles at the same temperature.

### 4.2. Particle Morphology

Manipulating the physical and chemical properties of a material in nanoscale can revolutionize electronic, diagnostic, and therapeutic applications [140]. To prepare for potential large-scale use of nanomaterials, it is important to determine whether nanoscale materials display undesirable effects, such as toxicity. To interpret the results of cell culture and animal models, nanomaterials should be thoroughly characterized with regard to the correlation between observed toxic reactions vis-à-vis the physical and chemical properties of the material. The structure of nanoparticles can be measured using techniques such as atomic force microscopy (AFM) and scanning electron microscopy (SEM) [145,146,147]. AFM or scan force microscope (SFM) is an extremely high resolution scanning probe microscope with a resolution of nanometer fractions, which is approximately 1000 times better than the optical diffraction limit. SEM is a type of electronic microscope wherein the images of the surface of a specimen are captured by examining the surface of a sample with a high energy electron beam in a raster injection pattern. SEM has the nanometer resolution required for the size of the submicron range, which is critical for determining particle shape. The electron interacts with the atoms of the sample, leading to the production of the signal that contains sample information, such as surface topography, composition, and electrical conductivity.

### 4.3. Surface Charge

Upon intravenous administration of nanoparticles, the immune system readily recognizes the foreign materials during the circulation process and then they are subsequently removed via the phagocytosis process [148]. Several factors are involved in the removal process, i.e., surface charge, hydrophobicity, and size of the nanoparticles. Therefore, many have studied ways in which to model the surface of the nanoparticles. By measuring surface charge, density, and surface hydrophilicity, the efficiency of surface modification can be predicted. One common way to know the surface charge is by measuring the zeta potential of nanoparticles in aqueous solutions. In addition, polydispersity index can be used to measure the distribution of the nanoparticles. Interaction between particles plays an important role in colloidal stability. Using the zeta potential measurement to predict stability quantifies this interaction [146]. Zeta potential is a measure of rebound between particles. In addition, since most water-soluble colloid systems are stabilized by electrostatic repulsion, the greater the repulsion between the particles, the less likely they are to come closer to each other and form cohesion [148]. Nanoparticles with jet potential of more than 30 mV (+/−) have been reported to be stable in the deposit because the surface charge prevents the particles from clotting. Zeta potential can likewise be used to examine whether the loaded active material is encapsulated within the center of the nanoparticle or adsorbed to the surface.

## 5. Loading and Release of Drug

### 5.1. Loading of Drug

For drug administration, the soluble spotting of nanoparticles should have a high drug-loading capacity with a reduced volume of media. The drug is loaded onto nanoparticles in two ways [149]. The first method is to combine (or add) nanoparticles with a drug when producing nanoparticles and to load them simultaneously. The second method is to attach or insert concentrated drug solutions to nanoparticles, such as drug absorption, after the synthesis of nanoparticles. Drug-loading effects vary depending on drug solubility, nanoparticle size, media materials, and polymers. Substance solubility of polymers linked to the composition, MW, and drug-polymer interaction of polymers play major roles. Moreover, the size of nanoparticles is proportional to the loading amount efficiency when proteins are loaded at or near the IEP [150]. Kim et al. studied the loading efficiency of curcumin-loaded HSA nanoparticles [151]. As a result, curcumin loading increased parallel with the curcumin concentration, while the ratio of organic solvents and water decreased. Optimal curcumin loading (7.2 ± 2.5%) was obtained at 2% (*w/w*) of HSA concentration, 50 mg/mL of curcumin concentration, and 1:19 ratio of organic solvents and water.

### 5.2. Drug Release

Drug release and polymer biodegradability are significant parameters of nanoparticles for successful drug delivery. Drug emissions are affected by the solubility of the drug, drug proliferation through the nanoparticle matrix, nanoparticle decomposition, and the erosion and diffusion processes [152]. The emission mechanism is dependent on solubility, diffusion, and nanoparticle biodegradation. In the case of concrete nanoparticles with uniformly distributed drugs, emissions are caused by the degradation or dispersion of the material. In addition, the dosing method likewise affects the release pattern. Thus, if the rate of diffusion is faster than that of the decomposition of the matrix, the emission becomes dependent mainly on the diffusion, otherwise it becomes dependent on the degradation of the encapsulation matrix. The release of drugs from protein-based nanoparticles can theoretically be considered, such as protein erosion or degradation, spread of drugs through pores, release from polymer surfaces, and pulse delivery through an electrified magnetic or sonic field [153]. Usually, emission studies are conducted by stirring and centrifugal control. Dialysis technology has been commonly used for the separation process from the emission media. Duclairoir et al. measured the release of various polar drugs from the gliadin nanoparticles [154]. The results showed that hydrophobic drugs in the gliadin nanoparticles are released slower than hydrophilic drugs due to the high affinity between the hydrophobic drug and the hydrophobic gliadin protein. In addition, hydrophilic drugs displayed a slow spread from nanoparticle matrices, following the burst release.

## 6. FDA Approval of Protein Nano-Drugs for Medical Purposes

Since 1995, 50 nano-pharmaceuticals have received FDA approval and are currently available for clinical use [155]. In most cases, nano-drugs are administered intravenously or orally. FDA-approved nano-drugs available for clinical use include liposome nanoparticles, polymer nanoparticles, micelle nanoparticles, nanocrystal nanoparticles, inorganic nanoparticles (metals/metal oxides and other inorganic nanomaterials), and protein nanoparticles. These nano-drugs have been approved for a variety of applications, including cancer treatment [156,157]. High number of FDA-approvals of nano-drugs were seen between 2001 and 2005, followed by a significant decrease after the financial crisis in 2008 and decrease in the R&D investment [158]. It should be noted that only two protein nanoparticles have been FDA-approved among the 50 nano-drugs [159]. One is Abraxane (Celgene), an albumin-bound paclitaxel nanoparticle that is used to treat breast cancer, NSCLC, and pancreatic cancer. The advantage of Abraxane is that it has improved solubility and targeted delivery to tumor. The second one is Ontak (Eisai), a Denileukin diftitox (engineered protein combining L-2 and diphtheria toxin) nanoparticle that is used to treat cutaneous T-cell lymphoma. The advantage of Ontak is that it has targeted T-cell specificity and lysosomal escape property. A possible reason for the presence of only two protein nano-drugs in the nanomedicines approved by the FDA list is that albumin has its own endocytosis route mediated by albumin receptor gp60, located at caveolae [87]. With regard to cells that lack or have limited caveolae, additional ligands are required to efficiently deliver albumin nanoparticles loaded with therapeutics into target cells. Another reason could be due to the non-specific delivery of the therapeutics to non-targeted cells that have albumin receptor. In addition, maintaining the activity of drug cargo is also challenging since enzyme cargo might be deactivated during the nanoparticle preparation process (desolvation method).

## 7. Conclusions

Protein nanoparticles are delivery carriers and have various advantages and applications in the delivery of materials, such as genetic materials, anticancer drugs, peptide hormones, growth factors, DNA, and RNA. Protein nanoparticles have the advantages of being more stable and easier to manufacture, as compared with other colloidal carriers. In addition, high potential utilization in vivo is expected as protein from various sources can be manufactured into nanoparticles using an easy, cost-effective, and eco-friendly synthesis process, accompanied by the use of less chemicals, as compared with nanoparticles from other materials. Protein nanoparticles have their own pros and cons depending on the different materials and processes. Among the various proteins for drug delivery applications, fibroin and albumin are most widely used. On the other hand, the investigation on the use of legumin and proteins have begun in order to determine whether they are viable alternatives for drug delivery applications and whether there are problems to be overcome as well.

Methods such as desolvation and complex coacervation are often used in the nanoparticle production processes, however, nano spray drying is still in its beginning stage and other physical and chemical processes also have limitations, such as low flow rates or the need to remove surfactants. Therefore, more research should be actively carried out to overcome these limitations. Protein nanoparticles can improve protein transfer efficiency by controlling characteristics such as size, shape, and surface charge. Moreover, the protein drug loading and release efficacy are also regulated according to the characteristics of the nanoparticles or the concentration and type of the drug.

Although the application of protein nanoparticles already has some interesting results and has shown great potential in the future, comparative data on the performance and treatment efficiency of protein nanoparticles and other existing delivery systems are still scarce. Therefore, more research should be carried out. In order to manufacture efficient protein nanoparticles, the optimal material or process must be chosen on a case-to-case basis.

## Figures and Tables

**Table 1:** Advantages and disadvantages of each protein nanoparticles.

Material	Advantage	Disadvantage
Silk protein fibroin	High stability Flexibility with high mechanical strength, suitable for various machining conditions Low immunogenicity Biodegradability Biocompatibility	Sericin may cause immunogenic reactions Slow degradation of silk II crystalline antiparallel β-sheet domains
Human serum albumin	High stability High solubility in physiological fluids Biodegradability Non-immunogenicity Non-toxic Availability and readiness	Expensive cost
Gliadin	Biocompatibility Biodegradability Non-immunogenicity Non-toxic High stability	Large particle size Rapid degradation speed
Gelatin	Biocompatibility Biodegradability Ease of bridge Safety	Low mechanical strength Rapid degradation speed
Legumin	Bioadhesive Wide surface area Small particle size Low immunogenicity High stability	Low yield
30Kc19	High stability Increased cell growth and viability Biodegradability Non-immunogenicity Non-toxic Enzyme-stabilizing property Cell-penetrating property	Low nanoparticle size and yield when using only 30Kc19α
Lipoprotein	Non-immunogenicity Biodegradability Biocompatibility Long circulation half-life Naturally targeting property	Difficult to separate native LDL
Ferritin	High stability pH stability Thermal stability Biodegradability	High cost

**Table 2:** Advantages and disadvantages of each protein nanoparticle generation methods.

Method	Advantage	Disadvantage
Emulsion/solvent extraction	High stability The shape and size of nanoparticles can be controlled by reaction conditions High encapsulation efficiency	Generating particles larger than those obtained by desolvation Thermodynamic instability → Need surfactants and stabilizers
Polyelectrolyte complexation/complex coacervation method	High stability Small nanoparticles Can be mixed with sensitive drugs (protein or peptide) The shape and size of nanoparticles can be controlled by reaction conditions	Difficulty of scale-up
Electrospray technique	High stability Small nanoparticles Scalable at industry-level and already in use	Low flow This technique may induce some macromolecule degradation due to the stress involved in the operation parameters (e.g. Thermal stress in drying, shear stress in the nozzle).
Nano spray drying	Control of particle size, shape, and morphology One-step semi-continuous process Processing of heat-sensitive substances with low risk of degradation Cost-effective	Limited to small-scale production Challenging to incorporate hydrophobic drugs
Desolvation method	High stability Simple to manufacture Small nanoparticles High encapsulation efficiency The shape and size of nanoparticles can be controlled by reaction conditions.	Only possible for proteins that can be minimally affected by the de-soluble process itself or diluted by transporter proteins
Self-assembly	High encapsulation efficiency Small nanoparticles High stability	Difficult to control the size and shape of nanoparticles. Protein strain potential exists
